# Examining the Efficacy of a Telehealth-Based Virtual Reality Clinic in Treating Adults With Specific Phobia: Feasibility Randomized Controlled Trial

**DOI:** 10.2196/84670

**Published:** 2026-06-15

**Authors:** Brian E Bunnell, Kaitlyn R Schuler, Jason G Craggs, Brandon M Welch, Triton Ong

**Affiliations:** 1 Department of Psychiatry and Behavioral Neurosciences, Morsani College of Medicine, University of South Florida, 3515 E Fletcher Ave, Tampa, FL, 33613-4706, United States, 1 8139748607; 2 Department of Psychology, University of North Carolina Wilmington, Wilmington, NC, United States; 3 Doxy.me Research, Doxy.me Inc., Charleston, SC, United States

**Keywords:** virtual reality, exposure therapy, anxiety, mental health, phobias, telemedicine, telehealth, telemental health, telehealth-based VR

## Abstract

**Background:**

Virtual reality (VR) has the potential to enhance telemental health care (TMH) by enabling accessible, engaging, and personalized treatment from home. Although VR is well-supported for in-person treatment, evidence for telehealth-based VR is limited. Moreover, no prior research has demonstrated the feasibility of therapists and clients conducting therapy sessions remotely within a shared (ie, multiuser) VR experience.

**Objective:**

The primary objective of this study was to evaluate the feasibility of conducting a randomized controlled efficacy trial (RCT) comparing exposure therapy delivered via Doxy.me VR (Doxy.me Inc)—a multiuser, telehealth-based VR app—to standard TMH in adults with clinically elevated fear of dogs, snakes, or spiders. A secondary objective was to preliminarily examine clinical (ie, specific phobia symptom severity) and treatment-related outcomes (ie, therapeutic alliance, client satisfaction, system usability, presence, cybersickness, and treatment fidelity).

**Methods:**

This study used a single-site, fully remote, parallel, feasibility RCT design. Participants were randomly assigned using a 1:1 ratio to receive 12 weekly exposure therapy sessions over 3 months, delivered via either standard TMH or Doxy.me VR. Assessments were conducted at baseline, each session, midtreatment, and posttreatment. All therapy sessions were audio recorded, 20% of which were randomly selected and rated for treatment fidelity. Feasibility benchmarks during the 12-month trial included: (1) enrolling 30 participants during Months 1‐9 of the trial; (2) collecting 70% of midtreatment self-report data; (3) collecting 70% of posttreatment self-report data; (4) collecting 70% of weekly self-report data; and (5) achieving treatment fidelity ≥80%. Between-group differences in clinical treatment-related outcomes also were examined preliminarily.

**Results:**

A total of 54 participants were enrolled between October 25, 2023 and July 26, 2024, and randomly assigned to the Doxy.me VR (n=29) and TMH (n=25) conditions, exceeding our recruitment target by 180%. Among the 30 participants targeted for completion, data were obtained from 29 (96.7%) at midtreatment and 28 (93.3%) at posttreatment. Participants completed 86.5% (180/208) of weekly self-report assessments. Treatment fidelity was 90% based on ratings of 41 session recordings. There were no significant between-group differences in clinical outcomes (all *P*>.05) or most treatment-related outcomes. However, client satisfaction improved significantly more for the Doxy.me VR condition compared to TMH (*P*=.04). Power analyses indicated that a sample of 160 participants would ensure adequate power for a fully powered trial.

**Conclusions:**

Telehealth-based, multiuser, synchronous VRET is feasible and shows preliminary evidence of efficacy. These findings are promising, and opportunities to improve procedures identified in this feasibility trial will directly inform the protocol for a fully powered RCT evaluating telehealth-based VR and its potential to improve treatment of mental health disorders.

## Introduction

### Background

The landscape of mental health care has changed substantially with the increased uptake of telehealth (ie, synchronous videoconferencing visits between a provider and patient) and telemental health care (TMH; ie, telehealth for the treatment and management of behavioral health conditions) in recent years [1-3]. TMH provides numerous advantages to traditional in-person care, such as improved outcomes, appointment completion, access to care, and satisfaction, as well as reduced costs and the ability to overcome common barriers to mental health care (eg, stigma, scheduling, and transportation) [4-8]. TMH usage has continued to grow postpandemic, accounting for over 70% of telehealth visits in the United States during 2023 [9]. Given this trajectory and a constantly evolving technology industry, innovative approaches to facilitating TMH will be of tremendous benefit [1,10,11].

Virtual reality (VR) provides immersive, computer-simulated 3D environments and objects that can be interacted with by a person wearing a head-mounted display and motion-sensing controllers. VR has documented efficacy for enhancing mental health treatments [10,12-16], particularly in the delivery of exposure therapy [17]. Exposure therapy, the gold standard treatment approach for anxiety disorders, involves repeated exposures to feared stimuli in a controlled and safe environment until that fear response and associated anxiety diminishes [18]. Exposure therapy via TMH often includes exposure to multimedia stimuli (eg, screenshared photos or videos), but clients’ affective responses to these exposure stimuli can be limited [19,20]. Another approach is to use interactive simulations for exposure exercises, termed VR-based exposure therapy (VRET). The VRET yields clinical results similar to traditional in-person exposure and provides unique benefits for controllable and repeatable sessions within a therapist’s office [21,22]. However, advances in consumer technologies have made it possible to conduct VRET remotely.

Delivering VRET over telehealth has the potential to provide an engaging and immersive treatment experience for patients [12]. Telehealth-based VR may also improve important treatment-related factors such as therapeutic alliance (ie, the extent to which a patient and therapist experience a collaborative and trusting relationship) [23-26] and telepresence (ie, the extent to which digital places, activities, and other individuals feel real) [15,27]. Experts agree that VRET and other VR-enhanced therapies can be appropriate and beneficial when delivered remotely, and more research is needed to understand their potential [28]. However, most research to date has examined VR-based mental health therapy delivered in person [29,30], while only a limited number of studies have investigated it when delivered remotely [31-33]. Moreover, no prior research has demonstrated the feasibility of therapists and clients conducting therapy sessions remotely within a shared (ie, multiuser) VR experience on an accessible consumer device.

### This Study

To address these limitations, we used insights gained from our research and engagement with mental health therapist and client stakeholders [13-15,34] to develop Doxy.me VR (Doxy.me), an innovative app for immersive, multiuser telehealth-based VR [35]. This study included a feasibility randomized controlled efficacy trial (RCT) comparing exposure therapy delivered via Doxy.me VR versus standard TMH to adults with a clinically elevated fear of dogs, snakes, or spiders. The primary aim of this study was to assess the feasibility of our study methodology and refine that methodology in preparation for a fully powered RCT. The secondary aim was to preliminarily examine clinical and treatment-related outcomes, including specific phobia symptoms, therapeutic alliance, and presence.

## Methods

### Trial Design

This study used a single-site, fully remote, parallel, feasibility RCT design and aimed to enroll between 40 and 60 adults with a clinically elevated fear of dogs, snakes, or spiders with the goal of completing treatment with 30 adults. Participants were randomly assigned using the REDCap (Research Electronic Data Capture; Vanderbilt University) [36,37] randomization module on a 1:1 allocation ratio to receive 12 weekly sessions of exposure therapy over the course of 3 months delivered via standard TMH versus Doxy.me VR. Study assessments were conducted at baseline, each session, midtreatment (ie, after completion of 6 therapy sessions, up to 6-wk postbaseline), and posttreatment (ie, 3 mo postbaseline). A comprehensive description of the protocol for this study is provided in a previous publication [15].

### Doxy.me VR

**Figure 1.**
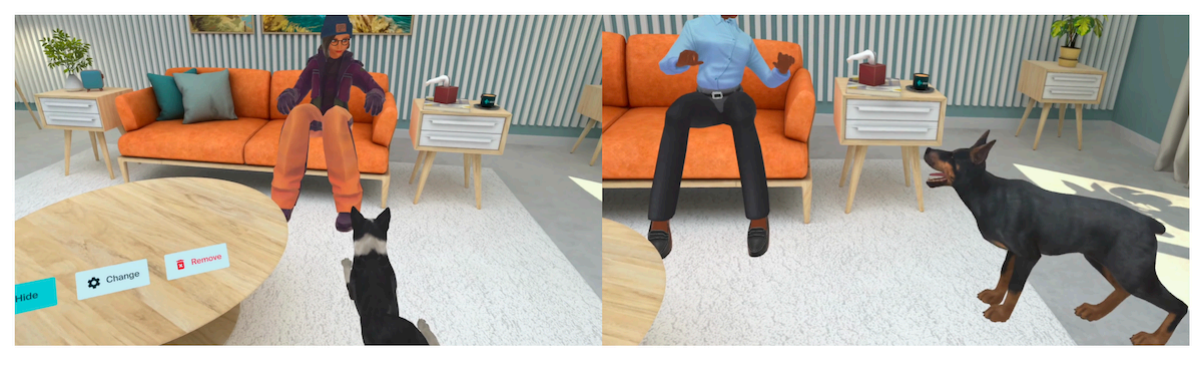
Provider helping clients tolerate a calm dog (left) and an aggressive dog (right).

Doxy.me VR is designed for a therapist to meet with a client remotely in a private, comfortable VR clinic that looks and feels like a therapist’s office (see Figure 1 for screenshots of the clinic). To join the VR session, the therapist provides their unique, persistent 4-digit room code to their client, who enters the code to check in. Once the therapist admits the client, they can interact by speaking and gesturing with each other in immersive VR. Therapists can use a menu to spawn animals such as dogs, snakes, and spiders for use in treating specific phobias. Multiple exemplars are available for each type of animal in varying sizes. For example, there are small dogs (eg, Chihuahua, Corgi, or Jack Russell Terrier), medium dogs (eg, Golden Retriever, Shiba Inu, or Pit Bull), and large dogs (eg, German Shepherd, Doberman, or Husky). Each animal is animated with four behavior states: idle (ie, no movement), calm (ie, small or slow movements in a relaxed posture), active (ie, fast or frequent movements in a playful posture), and aggressive (ie, fast or attacking movements in a defensive posture). Each behavior state also includes corresponding audio such as a calm dog breathing lightly, an active dog panting and barking playfully, and an aggressive dog snarling and barking loudly. Therapists can select, rotate, and move these animals around the room before making them visible to clients. Once therapists make the animal visible, they can continue to move and rotate the animal or remove the animal entirely. Clients cannot directly manipulate objects; however, clients gain access to all the control features while engaging with Doxy.me VR’s homework mode, which is used for between-session practice.

### Study Intervention

This study used an adapted version of the treatment protocol developed specifically for VRET by Bouchard et al [38], Côté and Bouchard [39], Michaliszyn et al [40], and St-Jacques et al [41]. After an initial baseline assessment, participants received 12 therapy sessions, each lasting up to 60 minutes. The treatment began with a single session focused on psychoeducation and planning (session 1), followed by 10 sessions dedicated to exposure therapy (sessions 2‐11), and concluded with one session on relapse prevention (session 12). In the first session, participants were introduced to the core principles of cognitive behavioral therapy, including the development of anxiety and specific phobias, as well as the rationale and process of exposure therapy. With guidance from the therapist, participants constructed a fear hierarchy, an ordered list of anxiety-provoking situations ranging from least to most distressing. These hierarchies incorporated text descriptions of VR or multimedia scenarios featuring static and dynamic representations of feared animals and real-life “in vivo” exposures, such as petting a dog or handling a snake in a pet store. Sessions 2 through 11 began with a review of the previous week’s homework, followed by an approximately 35-minute exposure exercise. During these exercises, participants rated their anxiety every 5 minutes on a scale from 1 to 100 (none to most extreme anxiety possible). They continued the exposure until they (1) achieved a 50% reduction in anxiety from their peak response after the stimulus was introduced, following which they would advance to the next exposure in their hierarchy; (2) expressed an alternative functional belief that was sufficiently strong to counter the association with perceived threat; or (3) 35 minutes had passed. After completing the exposure, the therapist facilitated a discussion to help participants reflect on the exposure, challenge and reframe maladaptive beliefs about the feared stimuli using cognitive restructuring, and practice box breathing techniques.

Participants randomized to the Doxy.me group were provided with a Meta Quest 2 VR headset preloaded with the Doxy.me VR app, to be returned to the study team following completion of the study. These participants also received a separate 30-minute training on how to use the headset and open the Doxy.me VR app. All therapy sessions were conducted via remote videoconferencing, with the Doxy.me VR group transitioning to the Doxy.me VR clinic for the exposure exercise portion of the session and the standard TMH group using multimedia (ie, photos and videos) screenshared by the study therapist during video calls. Once participants progressed through all situations in their hierarchy and were ready to complete their first in vivo exposure between therapy sessions for homework, weekly therapy sessions were devoted to processing and planning between-session in vivo exposures.

### Homework

All therapy sessions except session 12 included the assignment of homework exercises during the following week. These included reading an informational handout after the psychoeducation and treatment planning session (session 1) and exposure therapy assignments after therapy sessions 2‐11. Daily exposure-based homework assignments required participants to practice exposure exercises similar to those completed during that week’s therapy session (ie, using VR or multimedia stimuli) for a minimum of 30 minutes. For participants who stated they were ready to progress to in vivo exposures, these included in vivo exposure exercises planned during therapy sessions. Participants received SMS text messaging/email reminders with links to REDCap surveys that included instructions for each homework assignment and the situation to be used in the exposure exercise, which was entered by the therapist during the session. The survey asked participants to report the date and time of the exposure, their baseline anxiety rating, their peak anxiety level during the exposure, and their anxiety level following completion of the exposure exercise.

### Study Therapist Qualifications and Training

All participants were treated by one study therapist (KRS), who held a PhD in clinical psychology and was a postdoctoral scholar at the time the study was conducted. The study therapist had prior experience working on clinical trials and conducting exposure therapy. They had no prior experience using VR clinically or recreationally. Training was an iterative process as the treatment manual was being refined at the time but included the study therapist practicing study sessions with the principal investigator (BEB). The study therapist also participated in weekly clinical supervision with the principal investigator, who also listened to audio recordings of 20% of therapy sessions to rate treatment fidelity (see Fidelity to the Treatment Protocol). The Doxy.me VR development team was available to provide technical support to the study therapist as needed.

### Recruitment Strategy

Potential participants were recruited between October 25, 2023 and May 10, 2024 through Clinical Connection [42], ResearchMatch [43], Facebook advertisements [44], and flyers posted around the University of South Florida campus and off-campus community centers. Interested individuals were provided a URL or QR code linking them to an online prebaseline screening questionnaire hosted on REDCap. Those who met initial eligibility criteria were invited to schedule a consent and baseline assessment appointment using Microsoft Bookings, with automated text and email reminders sent 24 hours and 1 hour before the scheduled session.

### Eligibility Criteria

Eligible participants (1) were ≥18 years old; (2) had a self-reported fear of dogs, snakes, or spiders; (3) had Subthreshold or Present Specific Phobia symptoms as determined by the study therapist via administration of the Diagnostic Assessment Research Tool (DART) Specific Phobia Module [45]; (4) had access to the internet and a computer or smartphone with video conferencing capabilities; and (5) planned to reside in the state of Florida for the duration of the study. Individuals were ineligible to participate if they reported (1) participation in ongoing mental health therapy from a nonstudy therapist; (2) changes to psychotropic medication use within 6 weeks preceding enrollment in the trial; (3) active suicidal or homicidal intent or plan as determined by the study therapist via the DART Risk Assessment Module; (4) active auditory, visual, or tactile hallucinations via the DART Psychosis Module screening question; or (5) a diagnosis of photosensitive epilepsy by a medical doctor or a history of experiencing seizures caused by photosensitivity.

While we initially excluded individuals with a self-reported history of epilepsy or seizures, we modified this criterion 5 months into the trial to exclude individuals with a self-reported history of photosensitive seizures or belief that they had seizures resulting from photosensitivity. This change was made based on research suggesting that individuals with photosensitive seizures are at heightened risk of seizures during VR headset use, rather than individuals with a general history of seizures [45].

### Criteria for Withdrawing Participants

Participants were withdrawn from the study if they reported any of the following: (1) initiating therapy with a nonstudy mental health therapist; (2) changes in psychotropic medication use; (3) the emergence of active suicidal or homicidal thoughts or plans; (4) the onset of auditory, visual, or tactile hallucinations; (5) the development of photosensitive epilepsy or seizures; and (6) relocation outside the state of Florida during the study period. In cases of complete withdrawal—whether initiated by the participant or study team—study staff attempted to provide a referral to a local mental health provider. When contact was lost with participants after initiating treatment, study staff made efforts to administer midtreatment and posttreatment assessments. All participants were informed of their right to fully withdraw from the study at any time, including the option to request removal of previously collected data.

### Criteria for Wait-Listing Participants

If potential participants did not meet eligibility criteria due to ongoing mental health therapy with a nonstudy provider or recent changes in psychotropic medication use within the 6 weeks prior to enrollment, they were given the option to provide their contact information and be contacted by the study therapist to discuss their eligibility at a later date.

### Consent and Baseline Assessment

The consent and baseline assessment visit was conducted via a videoconferencing platform and was not audio- or video-recorded. During this visit, study staff: (1) reviewed and confirmed the potential participant’s responses from the online prescreening questionnaire; (2) provided detailed information about the study and obtained informed consent; (3) assisted participants in completing baseline questionnaires through a REDCap survey; (4) administered the specific phobia and risk assessment modules of the DART; and (5) made a final determination regarding the participant’s eligibility. Participants deemed eligible were then randomized using the REDCap randomization module and scheduled for their initial therapy session. Neither participants nor study staff were blinded to the treatment condition.

### Assessment Strategy and Measures

#### Overview

**Figure 2.**
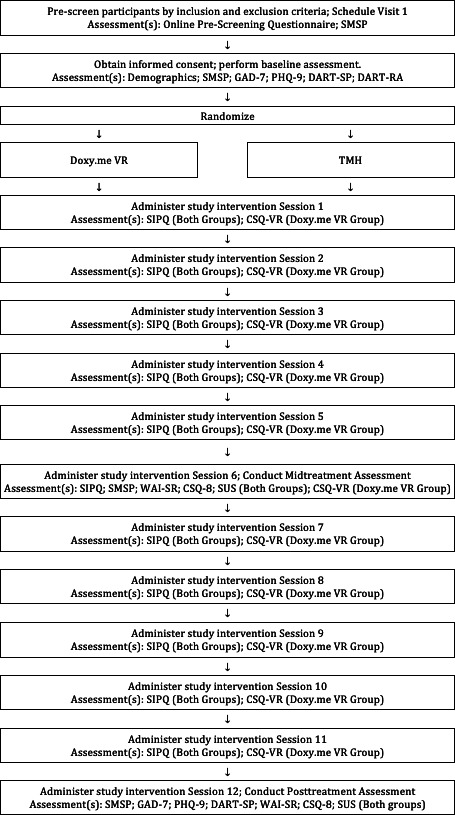
Study flow diagram. CSQ-8: Client Satisfaction Questionnaire; CSQ-VR: Cybersickness in Virtual Reality Questionnaire; DART-RA: Diagnostic Assessment Research Tool-Specific Phobia Module; GAD-7: Generalized Anxiety Disorder-7; PHQ-9: Patient Health Questionnaire-9; SIPQ: Single Item Presence Questionnaire; SMSP: Severity Measure for Specific Phobia; SUS: System Usability Scale; TMH: telemental health care; WAI-SR: Working Alliance Inventory-Short Revised.

Study assessments and their corresponding assessment time points are displayed in Table 1, and a diagram of the study flow is displayed in Figure 2. All assessments were conducted remotely by the study therapist and took place at baseline, during each therapy session, at midtreatment (ie, after completion of 6 therapy sessions, up to 6-wk postbaseline), and at posttreatment (ie, 3 mo postbaseline). Participants completed self-report questionnaires via REDCap, with the study therapist available to address any questions. Structured diagnostic interviews were administered by the study therapist and documented within REDCap. The primary outcome of the trial was specific phobia severity, as measured by the Severity Measure for Specific Phobia (SMSP) for adults [46].

**Table 1.** Study assessments and the timing of their administration during the study.

Domain	Measure	Time point				
		Pre	Baseline	Weekly	Mid	Post
Demographics	Demographics Questionnaire		✓			
Specific phobia severity	Severity Measure for Specific Phobia (SMSP)	✓	✓		✓	✓
Specific phobia diagnosis	Diagnostic Assessment Research Tool-Specific Phobia Module (DART-SP)		✓			✓
Risk	Diagnostic Assessment Research Tool-Risk Assessment (DART-RA)		✓			
Anxiety severity	General Anxiety Disorder-7 (GAD-7)		✓			✓
Depression severity	Patient Health Questionnaire-9 (PHQ-9)		✓			✓
Therapeutic alliance	Working Alliance Inventory-Short Revised (WAI-SR)				✓	✓
Presence	Single Item Presence Questionnaire (SIPQ)			✓		
Cybersickness	Cybersickness in Virtual Reality Questionnaire (CSQ-VR)			✓		
Treatment satisfaction	Client Satisfaction Questionnaire (CSQ-8)				✓	✓
Usability	System Usability Scale (SUS)				✓	✓

The SMSP [46] is a 10-item self-report questionnaire that assesses the severity of specific phobia symptoms relative to an individual’s feared stimulus. Total scores range from 0 to 4 with higher scores indicating higher phobia severity. The SMSP has excellent internal consistency, reliability, and criterion and discriminant validity [47].

#### Specific Phobia Diagnosis

The Diagnostic Assessment Research Tool (DART) [48] is a modular, semistructured interview assessing criteria of the *DSM-5* (*Diagnostic and Statistical Manual of Mental Disorders, Fifth Edition*) [49]. In this study, therapists administered the DART Specific Phobia Module (DART-SP), which classifies symptoms as absent, subthreshold, or present. The DART has demonstrated strong construct, discriminant, and convergent validity across its modules [48,50].

#### Other Mental Health Symptoms

The Generalized Anxiety Disorder-7 (GAD-7) [51] is a 7-item self-report measure that assesses the severity of general anxiety symptomatology. Scores range from 0 to 21, with higher scores indicating higher anxiety severity. The GAD-7 has demonstrated excellent internal consistency, reliability, and good convergent validity [52].

The Patient Health Questionnaire-9 (PHQ-9) [53] is a 9-item self-report measure that assesses the severity of depression symptomatology. Scores range from 0 to 27, with higher scores indicating higher depression severity. The PHQ-9 has demonstrated strong convergent validity and specificity for depression diagnosis, as well as excellent internal consistency and reliability [53].

The DART-Risk Assessment Module (DART-RA) [48] was administered by the study therapist to assess risk for suicidal and homicidal ideation, intention, and plans, and guide the development of safety plans as needed.

#### Treatment-Related Factors

The Working Alliance Inventory-Self Report (WAI-SR) [54] is a 12-item measure that assesses client-reported therapeutic alliance. Total scores range from 12 to 60, with higher scores suggesting a better therapeutic alliance. The WAI-SR includes 3 subscales: Goal (ie, the degree to which clients and therapists agree on goals of treatment), Task Agreement (ie, the degree to which clients and therapists agree on how to achieve those goals), and Bond (ie, the degree to which clients and therapists have developed a personal bond). Subscale scores range from 4 to 20, with higher scores suggesting better alliance within each domain. The WAI-SR has demonstrated good convergent validity and excellent internal consistency and reliability [55].

The Client Satisfaction Questionnaire-8 (CSQ-8) [56] is an 8-item client-rated questionnaire that assesses satisfaction with treatment. Scores range from 8 to 32, with higher scores indicating increased satisfaction. The CSQ-8 is correlated with clinical outcomes and posttreatment functioning and has demonstrated good construct validity and internal consistency and reliability [56].

The System Usability Scale (SUS) [57] is a 10-item self-report questionnaire that assesses the usability of a particular software system, platform, or app. Scores range from 0 to 100, with scores >68 indicating good usability, scores >80 indicating excellent usability, and scores <60 suggesting potential usability issues. The SUS has demonstrated good internal consistency and reliability [57].

The Single Item Presence Questionnaire (SIPQ) [58] is a 1-item self-reported measure of telepresence that asks respondents, “To what extent did you feel present in the environment, as if you were really there?” Ratings are provided on a scale of 0 “not at all present”-10 “totally present.” The SIPQ has demonstrated good to excellent validity, reliability, and sensitivity [58].

The Cybersickness in VR Questionnaire (CSQ-VR) [59] is a 6-item self-report questionnaire that assesses nausea, vestibular, and oculomotor cybersickness experienced in VR. This measure was completed by participants in the Doxy.me VR condition after each therapy session that included VR-based exposures. Total scores range from 6 to 27, with higher scores reflecting increased levels of cybersickness. The CSQ-VR has demonstrated excellent internal consistency and convergent validity [60].

### Fidelity to the Treatment Protocol

All therapy sessions were audio recorded, and 20% were randomly selected and rated by the principal investigator using a Treatment Fidelity Checklist that was developed based on the treatment manual.

### Feasibility of Study Methodology

We used well-established expert recommendations [61,62] to inform the development of the following predefined feasibility benchmarks during the 12-month trial: (1) enroll 30 participants during months 1‐9 of the trial; (2) collect 70% of midtreatment self-report data; (3) collect 70% of posttreatment self-report data; (4) collect 70% of weekly self-report data; and (5) achieve treatment fidelity ≥80%.

### Partial Automation of Trial Data Collection Using REDCap

All feasibility trial data collection and most of the trial components were automated using REDCap. This included administering the prebaseline screening questionnaire; obtaining informed consent via the REDCap e-Consent Framework feature; administering and calculating scores for all study questionnaires; randomizing participants via the randomization module; recording therapy session data (ie, session number, date, time, and length; adherence checklists; session notes; and in-session exposure ratings); and sending automated survey invitations and reminders for participants to complete homework assignments. A complete description and a figure displaying specific REDCap automations and features used for each stage of the trial are provided in a previous publication [15].

### Procedures to Ensure Participant Safety

Participants were informed that some questions asked during assessments and exposure exercises may cause distress, but that this distress was expected to be similar to what they would experience with routine care for specific fears/phobias. The study therapist was available to ensure that any questions causing distress were discussed with participants and provide them with techniques to reduce distress. Participants were also informed that a temporary increase in the severity of anxiety was to be expected at the beginning of exposure therapy, along with a gradual decline in severity over the course of treatment. Participants in the Doxy.me VR group were also informed that cybersickness was possible as a result of engaging with the VR environment and were asked to inform the study therapist if they began to feel nauseous or dizzy during therapy sessions so that a 5-10 minute break could be taken to allow symptoms to resolve. If participants were distressed and unable to participate in the study following baseline assessments, or if at the end of the study participants felt that they needed further treatment, the study therapist was available to provide a referral to therapists in the participants’ local area. Referrals were to be provided verbally to participants, and the study therapist was to follow up in one week via telephone to inquire as to the state of the referral and provide further assistance as necessary. The standard procedures in the event that participants endorsed suicidality or homicidality at any point during the study included the study therapist developing a safety plan with the participant and following up with that participant each session. The standard procedures in the event that participants endorsed imminent risk of harming themselves or others included the study therapist notifying the principal investigator (BEB) to conduct further risk assessment and contact emergency services if necessary.

### Ethical Considerations

The protocol was approved by the University of South Florida Institutional Review Board (#006215). All participant data were stored in the University of South Florida REDCap database, and only study staff members had access to the REDCap project. All data exported for analysis from REDCap were anonymized using participant study ID numbers. Participants were compensated US $50 for completing each baseline, midtreatment, and posttreatment assessment, for a total of US $150. Participants were compensated using e-gift cards.

### Data Analytic Plan

The primary goal of this study was to assess the feasibility of the study methodology, the results of which are presented descriptively. Chi-square tests of independence were used to assess associations between conditions and categorical variables. An independent sample *t* test and ANOVA were used to assess for significant differences between groups for continuous variables measured at one time point. Repeated Measures Analyses of Variance (rANOVA) were used to assess for significant differences between groups for continuous variables measured at multiple time points. Given the small sample size and that the primary focus of this trial was to assess feasibility, adjustments for multiple comparisons were not applied. As such, findings from inferential analyses should be interpreted as descriptive. All statistical analyses were conducted using SPSS (IBM Corp) [63]. G*Power (Heinrich Heine University Düsseldorf) [64] was used to conduct power analyses to inform sample size estimates for a fully powered trial.

## Results

### Recruitment

**Figure 3.**
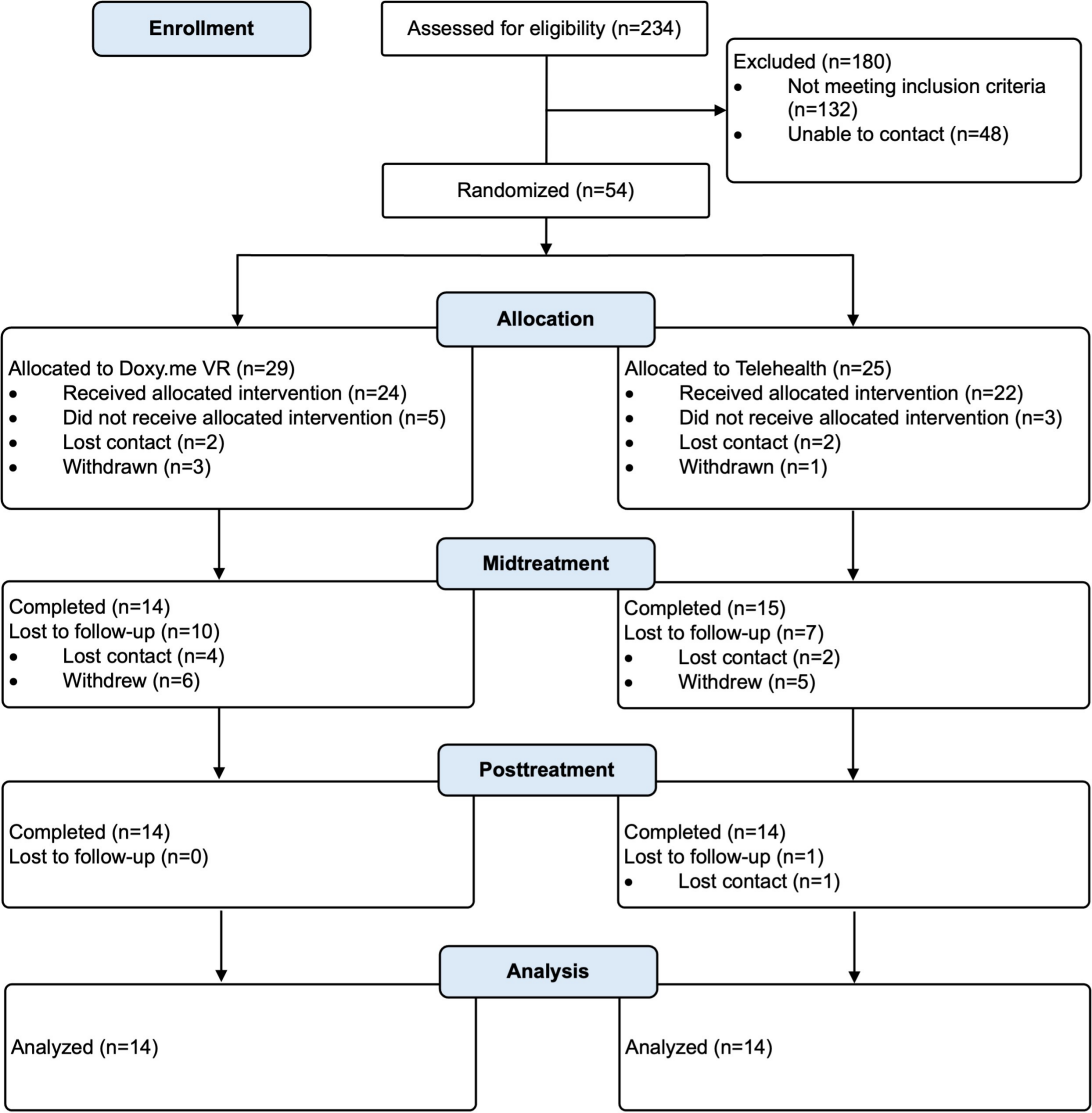
CONSORT (Consolidated Standards of Reporting Trials) flow diagram.

Participant recruitment and follow-up are displayed in Figure 3. The first participant was enrolled on October 25, 2023, and the last therapy session and posttreatment assessment were completed on July 26, 2024. Of the 234 individuals assessed for eligibility, 54 were enrolled and randomized to the Doxy.me VR (n=29) and TMH (n=25) conditions. Of these, 29 completed midtreatment assessments and 28 completed posttreatment assessments. The main reasons for participant withdrawal from the study included economic (eg, housing and internet access), legal, and health-related complications.

### Sample Characteristics

Sample characteristics are displayed in Table 2. A total of 54 individuals completed the baseline session and were randomly assigned. The sample was comprised of 25 (46.3%) individuals in the TMH group and 29 (57.3%) in the Doxy.me VR group. The average age of each group was approximately the same (TMH=45.20 and Doxy.me VR=46.10). The overall sample was largely female (42/54, 77.8%), White (32/54, 59.3%), and non-Hispanic (46/54, 85.2%). Roughly half of participants endorsed fearing snakes (28/54, 51.9%) and spiders (28/54, 51.9%), while only 13.0% (7/54) reported fearing dogs. Participants in the 2 treatment conditions did not differ significantly in age (*F*_1,52_=.054; *P*=.89; η²_p_=.001), gender (*χ*^2^_54_=1.18; *P*=.55), or race (*χ*^2^_54_=1.28; *P*=.73), ethnicity (*χ*^2^_54_=3.11; *P*=.08).

**Table 2.** Sample characteristics.

Demographic variable	TMH^a^ (n=25)	Doxy.me VR^b^ (n=29)	Total (n=54)
Age (year), mean (SD)	45.20 (13.98)	46.10 (13.35)	45.69 (13.53)
Gender, n (%)			
Women	19 (76.0)	23 (79.3)	42 (77.8)
Men	6 (24.0)	5 (17.2)	11 (20.4)
Nonbinary/gender fluid	0 (0.0)	1 (3.4)	1 (1.9)
Race^c^, n (%)			
White	14 (56.0)	18 (62.1)	32 (59.3)
Black/African American	7 (28.0)	8 (27.6)	15 (27.8)
Multiracial	3 (12.0)	3 (10.3)	6 (11.1)
Asian	1 (4.0)	0 (0.0)	1 (1.9)
American Indian/Alaska Native	0 (0.0)	0 (0.0)	0 (0.0)
Native Hawaiian/Pacific Islander	0 (0.0)	0 (0.0)	0 (0.0)
Ethnicity^c^, n (%)			
Hispanic/Latino	6 (24.0)	2 (6.9)	8 (14.8)
Non-Hispanic/Latino	19 (76.0)	27 (93.1)	46 (85.2)
Phobia, n (%)			
Dog	2 (8.0)	5 (17.2)	7 (13.0)
Snake	13 (52.0)	15 (51.7)	28 (51.9)
Spider	13 (52.0)	15 (51.7)	28 (51.9)

a TMH: telemental health care.

b VR: virtual reality.

c Race and ethnicity were defined based on National Institutes of Health reporting guidelines.

### Session and Homework Data

Session and homework completion data are presented in Table 3. Participants attended an average of 5.52 (SD 3.48) sessions, with 85.8% (46/54) attending at least one session. Participants in the 2 conditions did not differ in the number of sessions attended (*F*_1,44_=.145; *P*=.70; η²_p_=.003) or the proportion that attended at least one session (*χ*^2^_54_=.292; *P*=.59). A total of 1482 homework exercises were assigned based on session attendance. The overall completion rate for homework exercises was 25.3% (375/1482) and participants in the 2 conditions did not differ significantly in the proportion of homework they completed, *t*_45_=.279 and *P*=.78. Of the participants who attended sessions, 34.8% (16/46) completed at least one in vivo exposure exercise for homework, and participants in the 2 conditions did not differ in the proportion who completed an in vivo exposure (*χ*^2^_54_=.698; *P*=.40). The average number of sessions attended until participants engaged in an in vivo exposure exercise for homework was 5.94 (SD 1.06), and the average number of weeks was 7.81 (SD 3.14). Participants in the 2 conditions did not differ in the number of sessions (*F*_1,14_=.448; *P*=.51; η²_p_=.031) or the number of weeks (*F*_1,14_=.005; *P*=.94; η²_p_=.000) until engaging in an in vivo exposure.

**Table 3.** Session and homework completion.

Session or homework measurement	TMH^a^ (n=25)	Doxy.me VR^b^ (n=29)	Total (n=54)
Sessions attended, mean (SD)	5.70 (3.23)	5.35 (3.74)	5.52 (3.48)
Attended one session, n (%)	22 (88.0)	24 (82.8)	46 (85.8)
Homework expected, n (%)	731 (100)	751 (100)	1482 (100)
Homework completed, n (%)	229 (31.3)	146 (19.4)	375 (25.3)
Completed in vivo exposure, n (%)	9 (40.9)	7 (29.2)	16 (34.8)
Sessions until in vivo, mean (SD)	5.78 (1.06)	6.14 (0.89)	5.94 (1.06)
Weeks until in vivo, mean (SD)	7.78 (2.13)	7.86 (1.68)	7.81 (2.13)

a TMH: telemental health care.

b VR: virtual reality.

### Clinical Outcomes and Treatment-Related Factors

Given that this feasibility trial was not powered to detect between-group differences, analyses of clinical outcomes and treatment-related factors were conducted to generate preliminary estimates of effects and variability rather than to test efficacy hypotheses.

#### Clinical Outcomes

**Figure 4.**
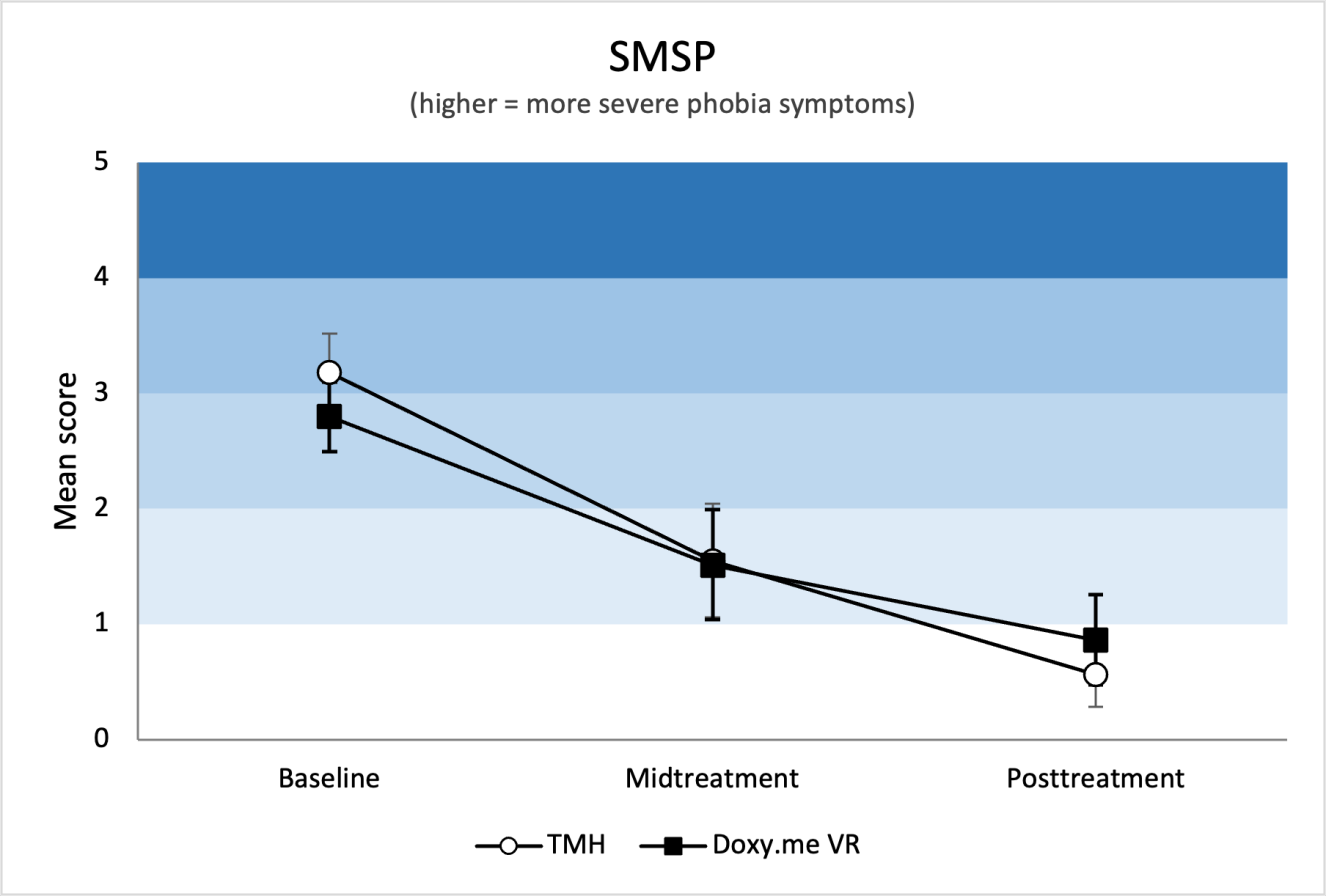
Mean Severity Measure for Specific Phobia (SMSP) scores across study time points. Error bars depict 95% CIs. SMSP: Severity Measure for Specific Phobia, TMH: telemental health care.

**Figure 5.**
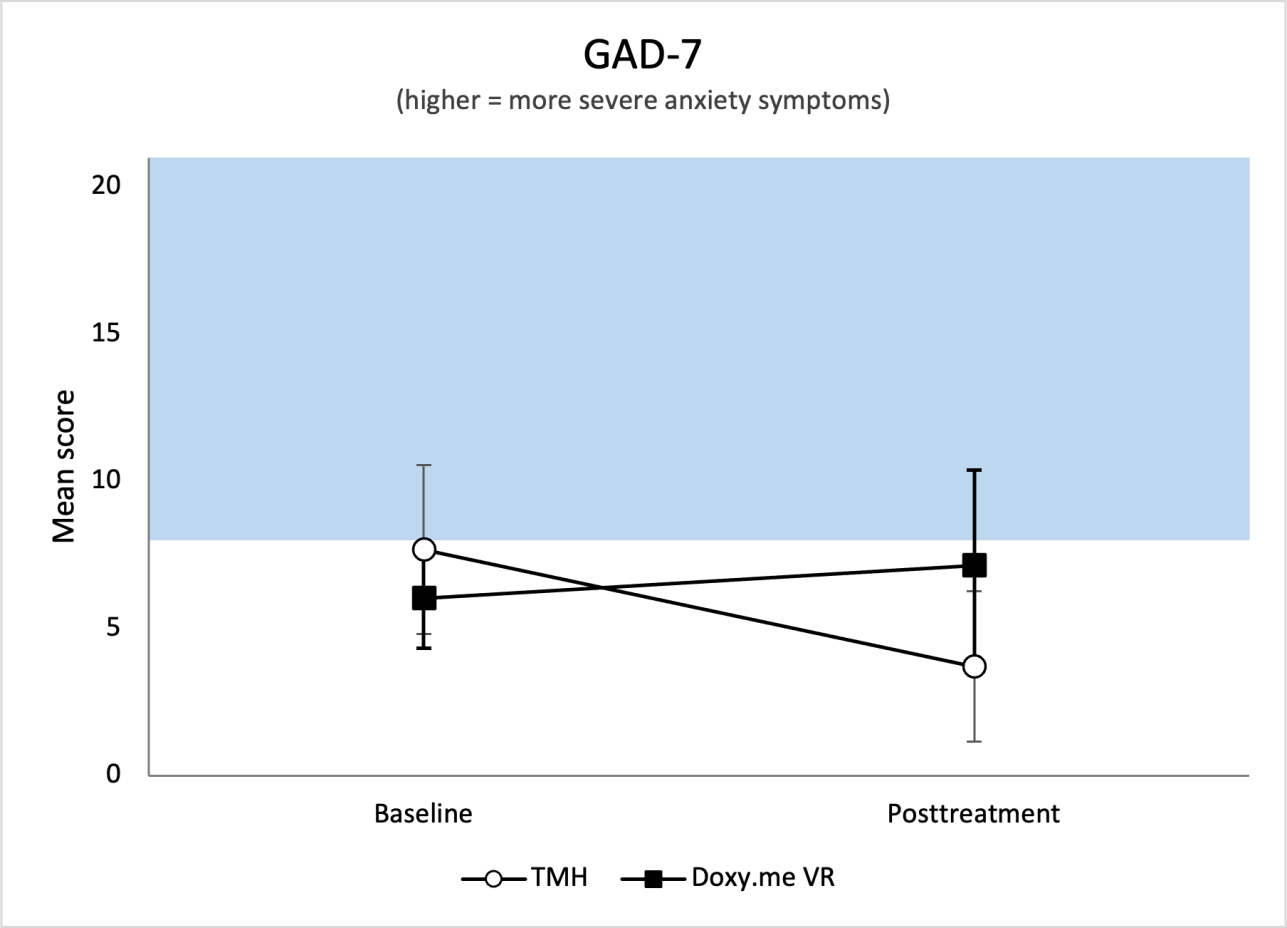
Mean General Anxiety Disorder-7 (GAD-7) scores across study time points. Error bars depict 95% CIs. GAD-7: General Anxiety Disorder-7; TMH: telemental health care.

**Figure 6.**
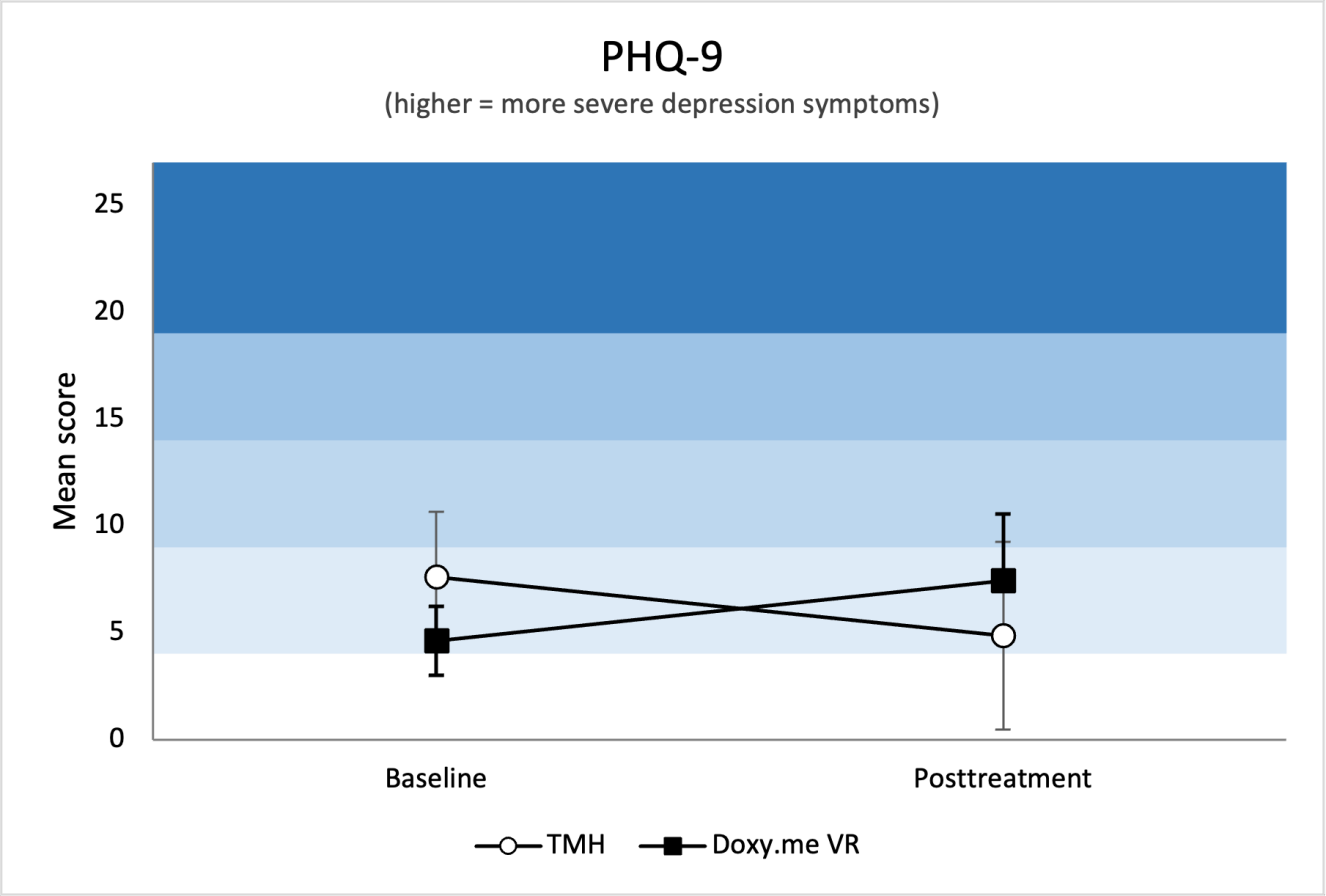
Mean Patient Health Questionnaire-9 (PHQ-9) scores across study time points. Error bars depict 95% CIs. PHQ-9: Patient Health Questionnaire-9; TMH: telemental health care.

Data for clinical outcomes and treatment-related factors are displayed in Table 4. Participants in both conditions exhibited decreased scores on the SMSP over time, and the results of the rANOVA indicated that the 2 conditions did not differ significantly on SMSP scores across the 3 assessment time points (*F*_2,26_=2.24; *P*=.18; η²_p_=.079; Figure 4). All participants met present or subthreshold criteria on the DART-SP at baseline, while 71.4% (15/21) of those who completed the DART-SP at posttreatment no longer met criteria (ie, designated as absent), and 23.8% (5/21) met subthreshold criteria. Participants in the 2 conditions did not differ significantly in the proportion that met present, subthreshold, or absent criteria on the DART-SP at baseline (*χ*^2^_54_=2.41; *P*=.12) or at posttreatment (*χ*^2^_54_=1.22; *P*=.54). Similarly, scores on the GAD-7 (Figure 5) and PHQ-9 (Figure 6) decreased over time, and the results of the rANOVA indicated that the 2 conditions did not differ significantly on the GAD-7 (*F*_1,26_=.66; *P*=.42; η²_p_=.025) or PHQ-9 (*F*_1,26_=1.14; *P*=.30; η²_p_=.042) from baseline to posttreatment.

**Table 4.** Clinical and treatment-related factor outcomes across study time points.

Measure	Baseline		Midtreatment		Posttreatment	
	TMH^a^ (n=25)	Doxy.me VR^b^ (n=29)	TMH (n=15)	Doxy.me VR (n=14)	TMH (n=14)	Doxy.me VR (n=14)
Specific phobia symptoms and severity, mean (SD)						
SMSP^c^ total	3.18 (0.82)	2.80 (0.80)	1.55 (0.89)	1.51 (0.83)	0.56 (0.47)	0.86 (0.68)
Specific phobia diagnosis, n (%)						
DART^d^-SP^e^ present	23 (92.0)	29 (100)	—^f^	—	0 (0.0)^d^	1 (10.0)^d^
DART-SP subthreshold	2 (8.0)	0 (0.0)	—	—	3 (27.3)^d^	2 (20.0)^d^
DART-SP absent	0 (0.0)	0 (0.0)	—	—	8 (72.7)^d^	7 (70.0)^d^
Other mental health symptoms, mean (SD)						
GAD-7^g^ Total	7.68 (6.95)	6.03 (4.48)	—	—	3.71 (4.41)	7.14 (5.63)
PHQ-9^h^ Total	7.60 (7.39)	4.62 (4.25)	—	—	4.86 (7.59)	7.43 (5.40)
Treatment-related factors mean (SD)						
WAI-SR^i^ total	—	—	57.67 (1.98)	55.64 (3.82)	57.21 (4.17)	56.93 (4.14)
WAI-SR Bond	—	—	19.73 (0.59)	19.14 (1.83)	18.86 (2.24)	19.14 (2.07)
WAI-SR Task	—	—	18.13 (1.55)	17.79 (1.25)	18.86 (1.35)	18.50 (1.70)
WAI-SR Goal	—	—	19.80 (0.41)	18.71 (1.77)	19.50 (0.94)	19.29 (1.43)
CSQ-8^j^ Total	—	—	31.47 (0.92)	30.36 (2.34)	31.07 (1.38)	31.43 (1.40)
SUS^k^ Total	—	—	90.33 (13.72)	76.43 (17.17)	88.39 (19.82)	80.89 (11.99)
SIPQ^l^ total^m^	—	—	7.69 (2.04)	8.38 (1.11)	—	—
CSQ-VR^n^ total	—	—	—	8.38 (3.25)	—	—

a TMH: telemental health care.

b VR: virtual reality.

c SMSP: Severity Measure for Specific Phobia.

d Due to participant scheduling availability, the DART was administered to 10 (71.4%) Doxy.me VR participants and 11 (78.6%) TMH participants at posttreatment.

e DART-SP: Diagnostic Assessment Research Tool–Specific Phobia.

f Not applicable.

g GAD-7: Generalized Anxiety Disorder-7.

h PHQ-9: Patient Health Questionnaire-9.

i WAI-SR: Working Alliance Inventory-Short Revised.

j CSQ-8: Client Satisfaction Questionnaire-8.

k SUS: System Usability Scale.

l SIPQ: Single Item Presence Questionnaire.

m SIPQ Total and CSQ-VR Total were calculated as the mean across sessions.

n CSQ-VR: Cybersickness in Virtual Reality Questionnaire.

#### Treatment-Related Factors

**Figure 7.**
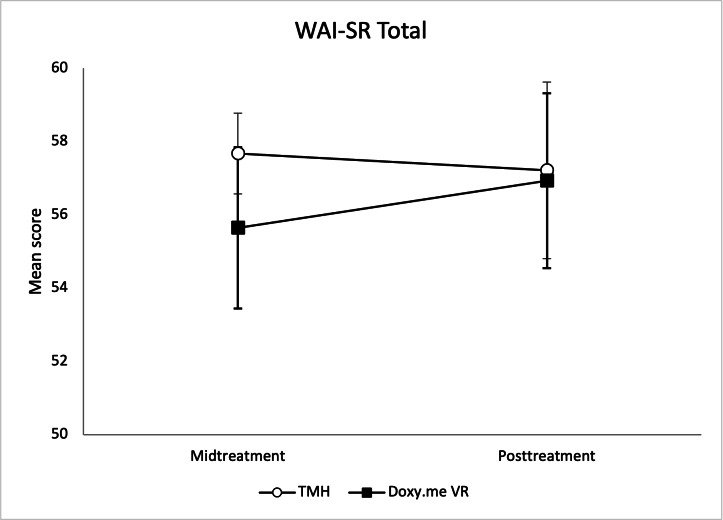
Mean Working Alliance Inventory-Short Revised (WAI-SR) total scores across study time points. Error bars depict 95% CIs. WAI-SR: Working Alliance Inventory-Short Revised; TMH: telemental health care.

**Figure 8.**
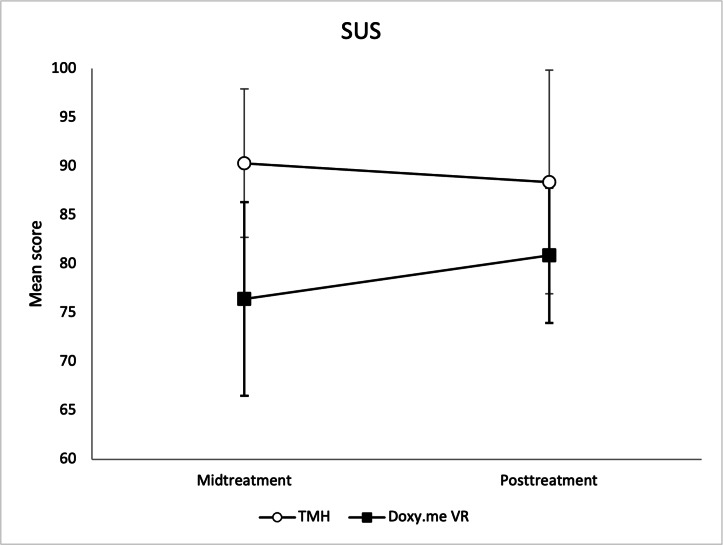
Mean System Usability Scale (SUS) scores across study time points. Error bars depict 95% CIs. SUS: System Usability Scale; TMH: telemental health care.

Scores on the WAI-SR remained consistent from mid- to posttreatment. The results of the rANOVA indicated that the 2 conditions did not differ significantly on WAI-SR Total (*F*_1,26_=1.79; *P*=.19; η²_p_=.065), Bond (*F*_1,26_=1.81 *P*=.19; η²_p_=.065; Figure 7), Task (*F*_1,26_=.00; *P*=.99; η²_p_=.000), and Goal (*F*_1,26_=3.02; *P*=.09; η²_p_=.104) scores from mid- to posttreatment. The results of the rANOVA indicated that the 2 conditions differed significantly on CSQ-8 scores (*F*_1,26_=4.64; *P*=.04; η²_p_=.151) from mid- to posttreatment, with the Doxy.me VR condition demonstrating a slight improvement in satisfaction compared to the TMH condition. Scores on the SUS remained fairly consistent from mid- to posttreatment, with the results of the rANOVA indicating that the 2 conditions did not differ significantly on SUS Total Scores (*F*_1,26_=2.99; *P*=.99; η²_p_=.103; Figure 8). Finally, the 2 conditions did not differ significantly on weekly averaged SIPQ scores (*F*_1,35_=.09; *P*=.76; η²_p_=.003).

### Fidelity to the Treatment Protocol

Fidelity to the treatment protocol was 90% based on ratings of a random sample of 41 (20%) of session recordings.

### Feasibility of Study Methodology and Lessons Learned

All benchmarks for feasibility were exceeded for this trial, and we identified opportunities to optimize the procedures (see Table 5). We exceeded our recruitment goal of 30 by enrolling 54 (180%) participants during Months 1‐9 of the trial. We obtained midtreatment self-report data from 29 (96.7%) and posttreatment self-report data from 28 (93.3%) of the 30 participants we aimed to complete the study with. Participants completed 180 out of 208 (86.5%) of weekly self-report assessments. Finally, fidelity to the treatment protocol was 90% based on ratings of a random sample of 41 (20%) of session recordings.

**Table 5.** Lessons learned and strategies for optimizing a fully powered trial.

This feasibility trial	Optimization for a fully powered trial
Recruitment and assessment	
Recruiting largely through Clinical Connection limited recruitment to individuals seeking compensated participation in clinical trials.	Partner with additional clinical trial advertising companies to recruit via community mental health groups and clinics.
Some participants appeared to malinger for incentives and seemed more likely to withdraw or disengage.	Include a malingering assessment at baseline to exclude participants simulating mental health symptoms.
Around 28% of participants withdrew due to economic (eg, housing and internet access), legal, and health-related complications.	Screen for health, housing, internet, and legal issues likely to interfere with participation prior to enrollment.
Around 46% of participants were withdrawn or lost contact.	Recruit 1.5x the sample required for final analyses.
Study coordination	
Budgetary limitations resulted in one study therapist managing most aspects of the trial (ie, setup, mailing, and troubleshooting virtual reality [VR] headsets, scheduling, compensation, and advertising).	Hire a full-time study coordinator to manage all study coordination tasks to enable study therapists to focus on providing the study intervention.
Intervention	
Participants found 12 sessions cumbersome, as most showed marked improvement within the first 6 sessions.	Reduce the number of sessions required from 12 to 7.
Some participants who enrolled were not retained past the first therapy session.	Improve retention strategies such as providing compensation for attending sessions.
VR technical and equipment problems	
Meta Quest updates changed underlying software infrastructure and caused VR sessions to be rescheduled or missed.	Set up Automated Testing to minimize downtime and fix issues before sessions.
Several sessions were delayed/rescheduled due to uncharged headsets and controller batteries.	Study coordinator will send participant reminders to charge headsets/controllers.
Around 20% (3/15) of VR headsets were not returned.	Budget for 20% more headsets than needed.
Protocol training and development	
Formal study intervention training was abbreviated and iterative due to time constraints and nature of the feasibility trial.	Conduct formal protocol training and regular fidelity checks to prevent protocol drift. Begin recruitment after study therapists reach fidelity.

### Power Analysis and Sample Size Estimation for a Fully Powered Trial

Based on the results of this feasibility trial, which showed effect sizes up to *d*=0.65 (η²_p_=.001 to .165), we conservatively assumed a group × time effect of *f*=0.25 for sample size estimation. Using G*Power, we conducted 2 power analyses:

- MANOVA (repeated measures, within-between interaction): *f*^2^(V)=0.25; *α*=.05; power=0.80, 2 groups, 3 time points → required n=158 (80 per group).
- ANCOVA (fixed effects with 2 covariates): *f*=0.25; *α*=.05; power=0.80, 2 groups → required n=158.

Thus, a sample of 160 (80 per group) ensures ≥0.80 power. To accommodate attrition, we will recruit 240 adults; even with 25% loss (~58), ≥86 per group will remain, exceeding the requirements. Based on prior experience, this sample is feasible to recruit and retain.

## Discussion

### Principal Results

This study examined the feasibility of conducting an RCT testing exposure therapy delivered via Doxy.me VR, a multiuser telehealth-based VR clinic, compared to standard TMH among adults with severe fears of dogs, snakes, or spiders. All a priori feasibility benchmarks were met or exceeded, and clinical symptom reductions were comparable across conditions. Treatment-related factors, including presence and therapeutic alliance, were also comparable, with client satisfaction improving significantly more in the Doxy.me VR condition.

In addition to demonstrating the feasibility of our study methodology, we gained valuable insights toward improving a future fully powered trial. First, our sample was enrolled largely through a single online source of recruitment (ie, clinical connection), which limited the recruitment pool to individuals seeking compensation for participating in clinical trials. This proved problematic as we lost contact with 18% and withdrew an additional 28% of participants, largely due to socioeconomic and health-related circumstances. Thus, we plan to use multiple recruitment sources to help diversify the recruitment pool in the fully powered RCT that follows this feasibility trial. We also plan to enroll 1.5 times the number recommended based on our power analysis (ie, n=160) to account for participant drop-off. Further, we plan to assess for malingering and screen for health, housing, internet, and legal issues prior to enrollment to prevent external interference with participation.

Our study logistics proved feasible for both participants and research staff. This was determined based on the ample amount of weekly, mid-, and posttreatment self-reported data obtained from participants and excellent treatment fidelity ratings of the study therapist. However, 20% of participants failed to return their VR headsets after the study. Some equipment loss is to be expected in clinical trials; thus, we will budget for additional headsets and consider device management platforms in future research. We also experienced technical VR issues that delayed or required rescheduling of sessions due to headsets not being charged and occasional networking errors. In future trials, we will send out reminders for participants to charge their headsets prior to their weekly appointments.

### Comparison With Prior Work

Numerous RCTs have compared VRET to null and positive controls [65,66]. However, these previous studies applied VRET either in-person with a therapist (eg, [67]), or as a self-led at-home intervention without direct interaction with a therapist (eg, [68]). To our knowledge, the current study is the first systematic demonstration of feasibility for therapists and clients interacting in a shared, remote VR experience on an accessible consumer device.

The phobia measures used in this study are well established; less so are measures of VR experience. Researchers are currently expanding and consolidating constructs such as immersion, presence, embodiment, and telepresence as elements of the VR experience [69,70]. There is growing evidence these measures may predict treatment outcomes and may become increasingly useful if refined for ease and practicality in clinical settings [71,72].

We found that phobia symptom severity, as measured by the SMSP, decreased substantially and approximately the same for both TMH and Doxy.me VR interventions. Meta-analyses have consistently found VRET and conventional exposure treatments to be equally effective for anxiety. While clinically significant, our planned larger RCT will be fully powered to detect statistical significance as well. Previous studies found that technical issues with VR equipment could negatively affect patient engagement [73]. We experienced such technical issues in this feasibility study and explored plans to minimize their impact in future research. Mobile device management platforms allow remote monitoring and updating of headsets to support troubleshooting by participants. We will also dedicate more time to training the study therapists, especially given that lack of formal training is the leading barrier to adoption of VR [74]. Collectively, the lessons learned in this feasibility trial will help ensure practical and successful implementation of a fully powered RCT.

### Limitations

This study has several limitations. First, as noted throughout this report, the primary aim of this study was to examine the feasibility of our trial methodology. Although preliminary analyses of within and between-group differences in clinical outcomes were reported, they should be interpreted with caution given the small sample size. Second, the use of a single study therapist limits the ability to account for therapist effects (eg, therapeutic style, alliance, and fidelity), and treatment fidelity was rated by the principal investigator and not blinded. To address these limitations, the fully powered RCT will include multiple therapists and independent fidelity raters to allow us to examine therapist-level effects. Third, comparing Doxy.me VR to conventional telehealth approaches allowed for a broad comparison of outcomes but lacked insight into mechanisms of change. Sham VR controls, in which participants experience VR with inactive intervention components, are common in modern clinical research [75]. An example of sham VR for the current study may have been for participants to view flat, 2D videos of small animals alone in VR, which would have allowed comparison with the interactive and immersive components of Doxy.me VR. However, such VR functionality was out of scope for this project, and we chose to highlight the additive benefits of VR and replicate exposure procedures that have become common since the recent popularization of telehealth [76]. It will be important for future research to dismantle the various effects of remote, multiuser VR-based mental health treatments to understand their specific benefits compared to self-led or in-person variations. Fourth, there is justifiable concern about user privacy with consumer devices such as the Meta Quest 2. Nevertheless, the ubiquity and usability of consumer VR devices make them important to study for use in health care. Health data policies such as the Health Insurance Portability and Accountability Act (HIPAA) and General Data Protection Regulation (GDPR) permit privacy exceptions with transparent and documented consent from patients [77,78]. It would be ideal to use VR devices designed for use in health care settings, including strict security and privacy features. Researchers, clinicians, and patient advocates should collaborate with industry to achieve these goals.

### Conclusions

Telehealth-based, multiuser, synchronous VRET is feasible and preliminarily efficacious based on data from this study. These findings are promising, and opportunities to improve procedures identified in this feasibility trial will directly inform the protocol for a fully powered RCT evaluating telehealth-based VR and its potential to improve treatment of mental health disorders. Ultimately, this line of research will extend the established utility of VRET, facilitate collaboration between industry and clinical care, and lead to further research on the clinical utility of remote multiuser VR.

## Supplementary material

10.2196/84670 Checklist 1 CONSORT-EHEALTH (Consolidated Standards of Reporting Trials of Electronic and Mobile Health Applications and Online Telehealth) checklist.

10.2196/84670 Checklist 2 Fidelity to the treatment protocol.

## Abbreviations

ANCOVA: analysis of covariance
CONSORT: Consolidated Standards of Reporting Trials
CSQ-8: Client Satisfaction Questionnaire-8
CSQ-VR: Cybersickness in Virtual Reality Questionnaire
DART: Diagnostic Assessment Research Tool
DART-RA: Diagnostic Assessment Research Tool – Risk Assessment Module
DART-SP: Diagnostic Assessment Research Tool – Specific Phobia Module
DSM-5: Diagnostic and Statistical Manual of Mental Disorders, Fifth Edition
GAD-7: Generalized Anxiety Disorder-7
HIPAA: Health Insurance Portability and Accountability Act
MANOVA: multivariate analysis of variance
PHQ-9: Patient Health Questionnaire-9
rANOVA: repeated measures analysis of variance
RCT: randomized controlled trial
REDCap: Research Electronic Data Capture
SIPQ: Single Item Presence Questionnaire
SMSP: Severity Measure for Specific Phobia
SPSS: Statistical Package for the Social Sciences
SUS: System Usability Scale
TMH: telemental health care
VR: virtual reality
VRET: virtual reality–based exposure therapy
WAI-SR: Working Alliance Inventory – Short Revised
