# Supplementary material for: Examining the Efficacy of a Telehealth-Based Virtual Reality Clinic in Treating Adults With Specific Phobia: Feasibility Randomized Controlled Trial
Source: JMIR Form Res. 2026 Jun 15;10:e84670. doi: 10.2196/84670 (PMC13268261; doi:10.2196/84670)
Supplement: Checklist 2 [file formative-v10-e84670-s002.docx]

# Multimedia Appendix 2: Treatment Fidelity Checklist

**Session 1: Psychoeducation & Treatment Planning**

| ****Completed**** | ****Agenda Item**** |
| --- | --- |
|  | Reviewed **assessment results and presented individualized case formulation** |
|  | **Provided overview of CBT principles and treatment process (i.e., what to expect)** |
|  | **Review the model of anxiety, emotions, and specific phobias** |
|  | **Discussed how anxiety is normal/adaptative but is a problem if it interferes with functioning** |
|  | **Discussed the role of avoidance in anxiety and specific phobias** |
|  | **Discussed the role of thoughts and beliefs in anxiety (i.e., interpretation and perception)** |
|  | **Introduce cognitive restructuring** |
|  | **Assisted participant in identifying cognitive distortions related to anxiety** |
|  | **Worked with participant to modify cognitive distortions related to anxiety** |
|  | **Introduce exposure and provide directions and procedures** |
|  | **Discussed how exposure therapy works and “rules of exposure” with the participant** |
|  | **Invited participant to provide suggestions on the exposure plan** |
|  | **Created a fear hierarchy with SUDS ratings; selected stimulus for the next session** |
|  | **Assign homework and plan next session** |
|  | **Assigned homework: Review session 1 handout** |

**Session 2: Initial In-Session Exposure**

| ****Completed**** | ****Agenda Item**** |
| --- | --- |
|  | **Review prior session and homework: Review Session 1 Handout** |
|  | **Provided positive reinforcement for any homework completion** |
|  | **Problem-solved barriers to homework completion, as needed** |
|  | **Assessed participant’s understanding of the treatment rationale and role of avoidance** |
|  | **Discussed the difference between therapeutic exposure and ordinary day to day confrontations with the feared stimuli** |
|  | **Conduct in-session exposure exercise** |
|  | **Asked participant for verbal ratings of anxiety (1-100%) at start of exposure and then every 5 minutes** |
|  | **Remained in current hierarchy step until (A) Anxiety was at an acceptable level (50% of peak after initial presentation of stimulus) OR (B) An alternative functional belief was sufficiently strong to counter the association with perceived threat.** |
|  | **Guided participant to the next step in the exposure hierarchy as necessary** |
|  | **Assessed for and corrected use of avoidance/safety behaviors as needed** |
|  | **If stimulus did not elicit a fear response, provided narrative or instructions to increase presence and quality of the exposure** |
|  | **Process exposure** |
|  | **Confirmed exposure was an adequate level of difficulty** |
|  | **Worked with participant to identify and resolve avoidance strategies** |
|  | **Identified and processed emotions related to the exposure** |
|  | **Used cognitive restructuring to reframe appraisals related to the exposure, as needed** |
|  | **Demonstrated and practiced breathing retraining and relaxation strategies** |
|  | **Assign homework** |
|  | **Assigned homework: Daily exposure exercise similar to in-session exercise** |
|  | **Assessed and problem-solved potential barriers to homework completion** |

**Sessions 3-11: In-Session Exposure**

| ****Completed**** | ****Agenda Item**** |
| --- | --- |
|  | **Review homework: Daily exposure exercise similar to in-session exercise** |
|  | **Provided positive reinforcement for any homework completion** |
|  | **Problem-solved barriers to homework completion, as needed** |
|  | **Confirmed exposure was an adequate level of difficulty** |
|  | **Identified and resolved avoidance strategies** |
|  | **Identified and processed emotions related to the exposure** |
|  | **Used cognitive restructuring to reframe appraisals related to the exposure, as needed** |
|  | **Conduct in-session exposure exercise** |
|  | **Ask participant for verbal ratings of anxiety (1-100%) at start of exposure and then every 5 minutes** |
|  | **Remained in current hierarchy step until (A) Anxiety is at an acceptable level (50% of peak after initial presentation of stimulus) OR (B) An alternative functional belief was sufficiently strong to counter the association with perceived threat.** |
|  | **Guided participant to the next step in the exposure hierarchy, repeat** |
|  | **Assessed for and corrected use of avoidance/safety behaviors** |
|  | **If stimulus was not eliciting a fear response, provided narrative or instructions to increase presence and quality of the exposure** |
|  | **Process exposure** |
|  | **Identified and resolved avoidance strategies** |
|  | **Confirmed exposure was an adequate level of difficulty** |
|  | **Identified and processed emotions related to the exposure** |
|  | **Used cognitive restructuring to reframe appraisals related to the exposure** |
|  | **Assign homework** |
|  | **Assigned homework: Daily exposure exercise similar to in-session exercise** |
|  | **Assessed and problem-solved potential barriers to homework completion** |

**Alternative Sessions 3–11: Planning and Processing Between-Session In-Vivo Exposures**

**When participant has completed all VR/Media hierarchy steps and states they are ready for in-vivo exposures.**

| ****Completed**** | ****Agenda Item**** |
| --- | --- |
|  | **Review homework: Daily exposure exercise similar to in-session exercise OR Daily in-vivo exposure exercise** |
|  | **Provided positive reinforcement for any homework completion** |
|  | **Problem-solved barriers to homework completion, as needed** |
|  | **Confirmed exposure was an adequate level of difficulty** |
|  | **Identified and resolved avoidance strategies** |
|  | **Identified and processed emotions related to the exposure** |
|  | **Used cognitive restructuring to reframe appraisals related to the exposure, as needed** |
|  | **Plan daily between-session in-vivo exposure exercises for the coming week** |
|  | **Progressed to first or next step of in-vivo exposure hierarchy** |
|  | **Identified opportunities for participant to conduct in-vivo exposure exercise (e.g., Friend or Family member with access to feared stimulus, local pet store, local zoo, college department with feared stimuli)** |
|  | **Established date and time to conduct exposure exercises** |
|  | **Assign homework** |
|  | **Assign homework: Daily in-vivo exposure exercise as planned** |
|  | **Assessed and problem-solved potential barriers to homework completion** |

**Session 12 – Relapse Prevention, and Closure (60 minutes)**

| ****Completed**** | ****Agenda**** |
| --- | --- |
|  | **Review homework: Daily exposure exercise similar to in-session exercise OR Daily in-vivo exposure exercise** |
|  | **Provided positive reinforcement for any homework completion** |
|  | **Problem-solved barriers to homework completion, as needed** |
|  | **Confirmed exposure was an adequate level of difficulty** |
|  | **Identified and resolved avoidance strategies** |
|  | **Identified and processed emotions related to the exposure** |
|  | **Used cognitive restructuring to reframe appraisals related to the exposure, as needed** |
|  | **Review treatment progress** |
|  | **Highlighted improvements in anxiety and reduction in avoidance behaviors** |
|  | **Summarized changes in stimuli-related appraisals** |
|  | **Reviewed skills the participant has learned** |
|  | **Reinforced participant’s self-efficacy to approach feared stimuli** |
|  | Create plan to strengthen and generalize treatment gains |
|  | **Discussed the need for continued self-guided exposure** |
|  | **Planned future self-guided in vivo exposures** |
|  | **Complete relapse prevention plan** |
|  | **Discussed difference between a lapse and relapse** |
|  | **Reviewed coping skills, such as cognitive restructuring and breathing retraining and identified barriers to practicing skills** |
|  | **Developed a plan for responding to lapses and relapses (i.e., resuming therapy, in vivo exposure, coping skills)** |
